# Diacerein modulates TLR4/ NF‐κB/IL‐1β and TRPC1/CHOP signalling pathways in gentamicin‐induced parotid toxicity in rats

**DOI:** 10.1111/jcmm.17791

**Published:** 2023-05-31

**Authors:** Dalia Mohamed Ali, Mohamed H. Mahmoud, Rehab Ahmed Rifaai, Michael Atef Fawzy, Medhat Atta, Nermeen N. Welson, Gaber El‐Saber Batiha, Athanasios Alexiou, Marios Papadakis, Walaa Yehia Abdelzaher

**Affiliations:** ^1^ Department of Forensic Medicine and Clinical Toxicology, Faculty of Medicine Minia University Minia Egypt; ^2^ Department of Biochemistry, College of Science King Saud University Riyadh Saudi Arabia; ^3^ Department of Histology and Cell Biology, Faculty of Medicine Minia University Minia Egypt; ^4^ Department of Biochemistry, Faculty of Pharmacy Minia University Minia Egypt; ^5^ Department of Anatomy, Faculty of Medicine Minia University Minia Egypt; ^6^ Department of Forensic Medicine and Clinical Toxicology, Faculty of Medicine Beni‐Suef University Beni Suef Egypt; ^7^ Department of Pharmacology and Therapeutics, Faculty of Veterinary Medicine Damanhour University Damanhour Egypt; ^8^ Department of Science and Engineering Novel Global Community Educational Foundation Hebersham New South Wales Australia; ^9^ AFNP Med Wien Austria; ^10^ Department of History of Medicine, School of Medicine University of Ioannina Ioannina Greece; ^11^ Department of Pharmacology, Faculty of Medicine Minia University Minia Egypt

**Keywords:** diacerein, gentamicin, parotid toxicity, rats

## Abstract

The present study aimed to identify the possible protective effect of diacerein (DIA) on gentamicin (GNT)‐induced parotid toxicity in rats. DIA was administered in the presence and absence of GNT. Thirty‐two Wistar adult male rats were randomly arranged into four groups: control, DIA (50 mg/kg/day), GNT (100 mg/kg) and GNT+DIA groups for 8 days. Parotid oxidative stress parameters, besides inflammatory and apoptotic biomarkers, were evaluated. Salivary flow rate, transient receptor potential canonical 1 (TRCP1), and C/EBP homologous protein (CHOP) in parotid tissue were measured. A parotid histopathological examination and an interleukin‐1 beta (IL‐1β) immunohistochemical study were also performed. GNT significantly increased parotid oxidative stress, inflammatory, apoptotic and CHOP biomarkers with decreased salivary flow rate and TRCP1 level. A histopathological picture of parotid damage and high IL‐1β immunoexpression were detected. DIA significantly normalized the distributed oxidative, inflammatory and apoptotic indicators, CHOP and TRCP1, with a prompt improvement in the histopathological picture and a decrease in IL‐1β immunoexpression. These results reported that DIA protects against GNT‐induced parotid toxicity via modulation of TLR4/NF‐κB/IL‐1β and TRPC1/CHOP signalling pathways.

## 1 INTRODUCTION

Gentamicin (GNT), a type of aminoglycoside, is considered the most vital antibiotic agent against a variety of species, particularly those that are resistant to other antibacterials. It is operative against gram‐negative and inadequate gram‐positive organisms. It is among the earliest and best‐known remedies for a number of bacterial diseases—such as bone, chest and urinary tract infections—pelvic inflammatory diseases, meningitis and sepsis. GNT is regarded by the WHO as being of vital importance for human medicine and is listed as one of the essential medicines.^1^

It functions by impeding the bacterial capacity to produce proteins, which usually results in bacterial death. Before being excreted in the kidneys via glomerular filtration, GNT is transported basically unaltered throughout the extracellular space instead of being metabolized. Potentially harmful side effects, most frequently ototoxicity^2^ and nephrotoxicity,^3^ place restrictions on its use. According to Abdollahi et al.,^4^ GNT may have an impact on the salivary glands.

In humans, there are 10 members of the large family of transmembrane recognition proteins known as Toll‐like receptors (TLR). It is crucial to the detection of pathogens and the activation of innate immunity. They are capable of identifying the pathogen‐associated molecular patterns that are expressed on infectious pathogens and facilitating the synthesis of the cytokines required for the maturation of functional immunity. TLR4 has recently been shown to be crucial for triggering the inflammatory response. When it is activated, an intracellular signalling pathway of proinflammatory cytokines, such as nuclear factor‐kappa B (NF‐κB), tumour necrosis factor (TNF), and interleukin‐1 (IL‐1), is stimulated.^5^

The human transient receptor potential canonical or classical (TRPC1) protein comprises functional nonselective cationic channels with high calcium permeability. These channels are widely expressed in different human tissues and cell types. By supplying the Ca^2+^ influx pathway or depolarizing the membrane potential, they are an important factor in the control of intracellular calcium.^6^ These ion channels are therefore prospective targets for drugs to treat diseases like cancer, epilepsy, pain, arthritis and cardiac remodelling.^7^

The acinar and ductal cells of the salivary gland are thought to secrete fluid mostly in response to TRPC1. When TRPC1 is damaged, the endoplasmic reticulum (ER) Ca^2+^ level drops, salivary gland cells die and C/EBP homologous protein (CHOP) expression rises.^8^

Diacerein (DIA), a novel analgesic, antipyretic and anti‐inflammatory medication created specifically for the treatment of osteoarthritis, is a derivative of anthraquinone. DIA works by preventing human monocytes from producing IL‐1. By triggering NF‐κB and mitogen‐activated protein kinase signalling, interleukin‐1, a pro‐inflammatory and pro‐apoptotic substance, stimulates the production of cytokines. DIA has been shown to significantly reduce inflammatory damage by downregulating the interleukin‐1 beta (IL‐1β) receptor,^9^ as well as inhibiting the production of nitric oxide inflammatory cytokines such as IL‐1β and TNF‐α and preventing the TLR4/NF‐κB‐mediated signalling pathways.^10^

Salivary glands have become a valuable research tool to investigate some fundamental issues in physiology, pathology and pharmacology, including protein synthesis, salt and water transport and autonomic nerves and receptor pharmacology.^11^ Even though GNT is frequently used in anti‐infectious therapy, its negative effects on the salivary glands have not received enough attention. As a result, we were curious to explore the potential protective role of DIA against the effects of GNT, a commonly used antibiotic, on the rat parotid glands, with a focus on the TRPC1/CHOP and TLR4 signalling pathways, as well as the likely mechanisms mediating its impact.

## 2 MATERIALS AND METHODS

### 2.1 Ethics

Rats' processing and therapy were done in accordance with the Institutional Ethical Committee's (Faculty of Medicine, Minia University, Egypt) guidelines for the care of experimental animals, as well as the National Institutes of Health's (NIH) Guide for the care and use of laboratory animals (Approval number: 398:2022).

### 2.2 Drugs and chemicals

DIA was purchased from EVA pharma, GNT, a pharmaceutical ampoules (20 mg) preparation, was purchased from Memphis Pharmaceutical & Chemical Industries, TRPC1 and CHOP ELISA kits were obtained from Wuhan Fine Biological Technology Co., Catalogue No. EH4284 and MyBioSource Co., Catalogue No. MBS3808179, respectively. Tumour necrosis factor alpha (TNF‐α) (Catalogue No. BMS622) ELISA kits were obtained from Thermo Fisher Scientific Inc. NF‐κB and Myd88 ELISA kits were purchased from ELISA Genie Co. Catalogue No: SKU: RTFI00988 and SKU: RTFI01303, respectively. Caspase‐3 ELISA kits from MyBioSource, Catalogue No. MBS261814. Total antioxidant capacity (TAC) commercial kits from Biodiagnostic, Catalogue No. TA 25 13.

### 2.3 Animals and experimental design

Thirty‐two male Wistar rats weighing 220–250 g, aged 7–10 weeks, were acquired from the National Research Center. Rats were left for acclimatization in their cages (4 rats/cage) in a normal light–dark cycle with free access to tap water and a normal diet (El‐Nile Company) for 10 days before initiating the experiment.

Animals were assigned into four experimental groups (8 rats/ group):

Group 1 (Control group): received vehicles.

Group 2 (DIA group): received diacerein 50 mg/kg/day for 8 days p.o.^12^

Group 3 (GNT group): received 100 mg/kg, i.p. for 8 days.^13^

Group 4 (GNT+DIA group): received 100 mg/kg, i.p., with diacerein 50 mg/kg/day, p.o for 8 days.

DIA dissolved in 1% carboxymethylcellulose (1% CMC) and freshly prepared before use.^12^

### 2.4 Sample collection

At the end of the experiment, the salivary flow rate was determined. Under the effect of anaesthesia with urethane hydrochloride (1 g/kg i.p.), salivation was stimulated by pilocarpine (1.0 mg/Kg body weight, i.p.), which was dissolved in distilled water, and the saliva that was produced was dripped in plastic tubes and maintained on ice during all the saliva collection period (40 min).^14^ Then, rats were sacrificed, and blood was collected from the abdominal aorta of the rats in heparinized syringes. It was then centrifuged at 4000 × ***g*** for 15 min (Janetzki T30 centrifuge). Then, sera were kept at −80°C for biochemical analysis. The parotid glands were excised and rinsed with saline to eliminate any blood. A part was fixed for histological studies. The other part was homogenized in ice‐cold phosphate buffer (0.01 M, pH 7.4; 20% w/v) with a glass homogenizer on ice (tissue weight (g): phosphate buffer (mL) volume = 1:5). The homogenate was centrifuged for 15 min at 5000 rpm, and the supernatant was stored at −80°C to measure the biochemical parameters.

### 2.5 Biochemical analysis

#### 2.5.1 Assessment of oxidative stress parameters

Malondialdehyde (MDA) is an index of lipid peroxidation. It was measured using the thiobarbituric acid method, and the sample absorption was determined at 535 nm.^15^

According to the previously prescribed method of Marklund and Marklund,^16^ one unit of superoxide dismutase (SOD) is equal to the amount of enzyme that prevents pyrogallol autoxidation by 50%. A spectrophotometer was used to measure enzyme activity at 420 nm.

TAC was estimated by colorimetric kits according to the manufacturer's instructions.

#### 2.5.2 Assessment of TRCP1 and CHOP levels

Parotid TRCP1 and CHOP levels were measured using ELISA kits, following the manufacturer's instructions. In CHOP, the colour of the tubes changed from blue to yellow, and the optical density was read at 450 nm within 15 min. In TRCP1, the colour turned yellow immediately, and the optical density was read at 450 nm after adding the stop solution.

#### 2.5.3 Assessment of inflammatory and apoptotic parameters

TNF‐, NF‐B, and caspase‐3 levels were measured using ELISA kits as directed by the manufacturer.

### 2.6 Western blotting of TLR4 and cleaved caspase‐3

Regarding western blotting, parotid gland homogenates (30 μg of total proteins) were heated for 5 min with loading buffer, then placed on 12% sodium dodecyl sulfate–polyacrylamide gel electrophoresis (SDS‐PAGE) which was then exposed to running at 100 V for 2 h. Separated proteins were carried to polyvinylidene fluoride (PVDF) membranes and subjected to blocking for 1 h in a Tris‐buffered saline (TBS‐T) blocking solution. Incubation with primary antibodies (1:1000) for rabbit anti‐TLR4 and anti‐C caspase‐3 antibodies (Catalogue No. ab13556, ab32042, Abcam, respectively) and β‐actin (Santa Cruz Biotechnology, Santa Cruz) overnight at 4°C then secondary antibody of polyclonal goat anti‐rabbit immunoglobulin (Cell Signaling Technology Inc.) was used at a dilution of 1:5000 in blocking buffer. Bands were seen by chemiluminescence and quantified using a luminescent image analyser (LAS‐5000, Fujifilm Co.). Bands of separated proteins were quantified after being normalized to β‐actin relative to the normal control group using ImageJ software.

### 2.7 Histological study

Specimens from the parotid gland were fixed, then dehydrated, cleared and embedded in paraffin. Sections with a thickness of 5 μm were cut and stained. Sections were examined blindly. The inflammation was scored from 0 to 3. A score of 0 indicates no inflammation; a score of 1 indicates mild inflammation with no foci; a score of 2 indicates moderate inflammation and one focus. A score of 3 indicates severe inflammation and more than one focus.^17^

### 2.8 Immunohistochemical staining

Immunohistochemistry for IL‐1β was performed on paraffin‐embedded tissue according to the manufacturer's protocol. Endogenous peroxidases were quenched by using 3% H_2_O_2_. Then sections were washed and incubated with monoclonal anti‐IL‐1β; Catalogue No. YMA1156 (1: 200) (Chongqing Biospes Co.). After washing with PBS, sections were incubated with the secondary antibody HRP Envision Kit (DAKO) 20 min, incubated with diaminobenzidine (DAB) for 10 min, and counterstained with haematoxylin. The mean area fraction for expression was measured using the image J‐22 program.

### 2.9 Statistical analysis

All values were expressed as mean ± SEM. The statistical analyses were conducted using GraphPad Prism (version 5.0). Analysis of variance (anova) for multiple comparisons, followed by the Tukey–Kramer test as a post‐anova test, was used for data analysis. The results were considered statistically significant if *p* < 0.05.

## 3 RESULTS

### 3.1 Effect of DIA on biochemical parameters in GNT‐induced parotid toxicity in rats

The GNT group showed significantly decreased parotid SOD and serum TAC with increased parotid MDA when compared to the control and DIA groups. Meanwhile, the GNT+DIA co‐treated rats showed significantly improved oxidative stress parameters when compared to the GNT group (Table 1).

**TABLE 1:** Effect of DIA on oxidative stress parameters in GNT‐induced parotid toxicity in rats.

Groups	Parotid MDA (nmol/g tissue)	Parotid SOD (U/g tissue)	Serum TAC (mmol/L)
Control	10.65 ± 0.79	291.80 ± 4.82	6.92 ± 0.33
DIA	11.16 ± 0.79	287.90 ± 6.81	7.50 ± 0.71
GNT	31.08 ± 1.64^a^ ^,^ ^b^	153.60 ± 3.78^a^ ^,^ ^b^	2.29 ± 0.20^a^ ^,^ ^b^
GNT+DIA	14.64 ± 1.05^c^	273.10 ± 6.01^c^	5.76 ± 0.46^c^

*Note*: Results represent the mean ± SEM (8 rats/group).

Abbreviations: DIA, diacerein; GNT, gentamicin; MDA, malondialdehyde; SOD, superoxide dismutase; TAC, total antioxidant capacity.

^a^ Significant (*p* < 0.05) difference from the control group.

^b^ Significant (*p* < 0.05) difference from the DIA group.

^c^ Significant (*p* < 0.05) difference from GNT.

**FIGURE 1:**
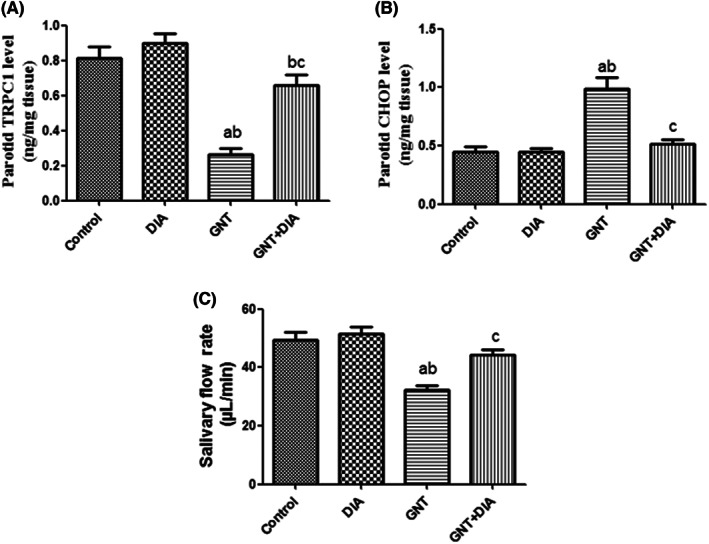
Effect of DIA on TRCP1, CHOP levels and salivary flowrate in GNT‐induced parotid toxicity in rats. (A) Effect of DIA on TRCP1, (B) Effect of DIA on CHOP, (C) Effect of DIA on salivary flow rate. Results represent the mean ± SEM (8 rats/group). ^a^Significant (*p* < 0.05) difference from the control group. ^b^Significant (*p* < 0.05) difference from the DIA group. ^c^Significant (*p* < 0.05) difference from GNT [CHOP, C/EBP homologous protein; DIA, diacerein; GNT, gentamicin; TRCP1, transient receptor potential canonical1].

In Figure 1, rats challenged with GNT revealed a significant decrease in salivary flow rate and parotid TRCP1 level and a significant increase in parotid CHOP level in comparison to the control and DIA groups. The GNT+DIA co‐treated rats showed a significant increase in salivary flow rate and parotid TRCP1 level and a significant decrease in parotid CHOP level when compared to the GNT group.

In Table 2, parotid TNF‐α, NF‐κB, and caspase‐3 levels significantly increased in the GNT group when compared to the control and DIA groups. On the contrary, the GNT+DIA group showed a significant decrease in the level of parotid TNF‐α, NF‐κB and caspase‐3 when compared to the GNT group.

**TABLE 2:** Effect of DIA on inflammatory and apoptotic parameters in GNT‐induced parotid toxicity in rats.

Groups	Parotid TNF‐α	Parotid NF‐kB	Parotid caspase‐3
	(pg/g tissue)	(ng/g tissue)	(ng/g tissue)
Control	51.42 ± 4.78	32.66 ± 2.94	10.73 ± 0.68
DIA	3.95 ± 50.73	33.84 ± 3.12	10.67 ± 0.66
GNT	177.60 ± 7.71^a^ ^,^ ^b^	80.19 ± 3.89^a^ ^,^ ^b^	33.90 ± 2.08^a^ ^,^ ^b^
GNT+DIA	63.43 ± 3.66^c^	36.10 ± 2.38^c^	12.47 ± 1.07^c^

*Note*: Results represent the mean ± SEM (8 rats/ group).

Abbreviations: DIA, diacerein; GNT, gentamicin; NF‐kB, Nuclear factor kappa B; TNF‐α, tumour necrosis factor alpha.

^a^ Significant (*p* < 0.05) difference from the control group.

^b^ Significant (*p* < 0.05) difference from the DIA group.

^c^ Significant (*p* < 0.05) difference from GNT.

**FIGURE 2:**
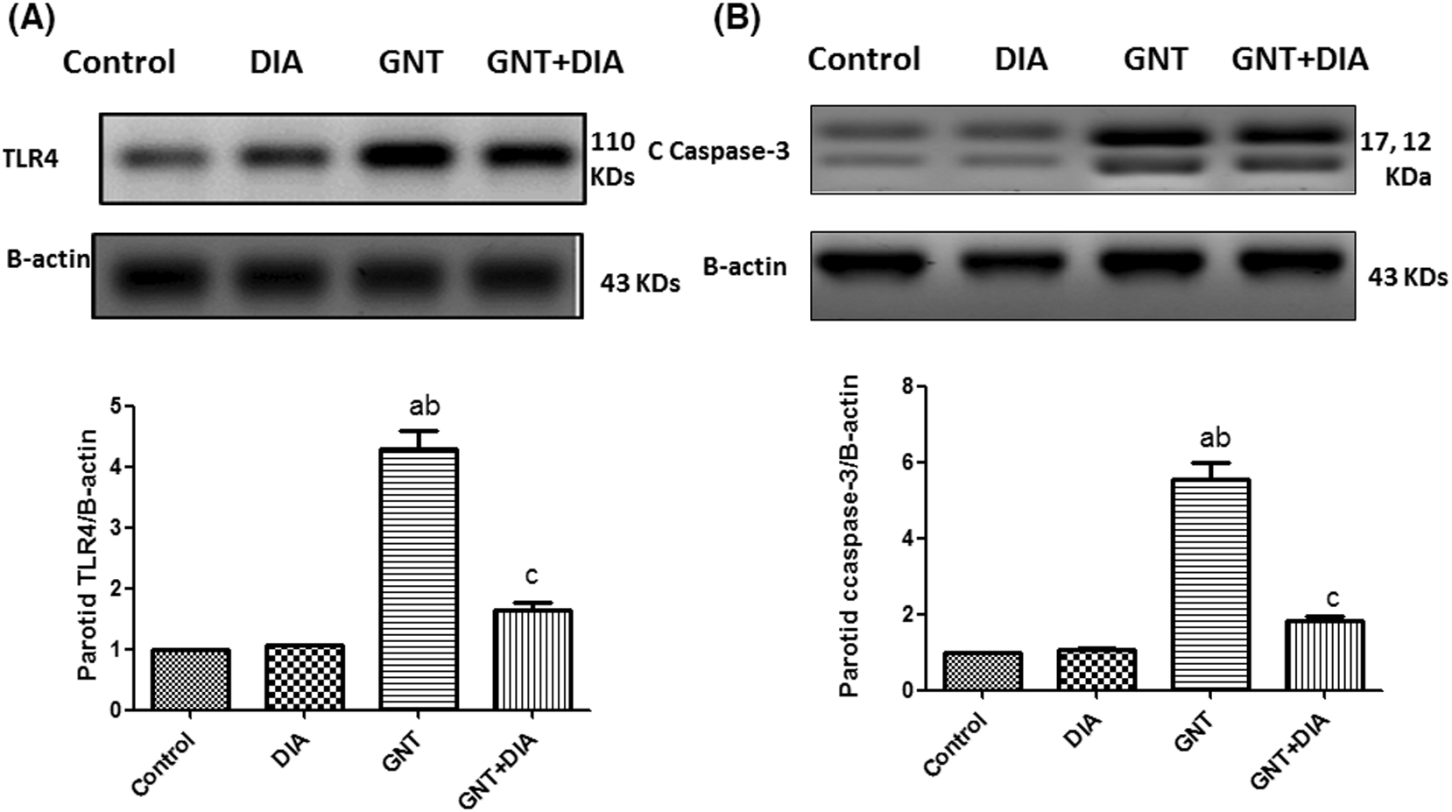
Effect of DIA on TLR4 and C caspase‐3 protein expressions in GNT‐induced parotid toxicity in rats. (A) Effect of DIA on TLR4, (B) Effect of DIA on C caspase‐3. Results represent the mean ± SEM (8 rats/group). ^a^Significant (*p* < 0.05) difference from the control group. ^b^Significant (*p* < 0.05) difference from the DIA group. ^c^Significant (*p* < 0.05) difference from the GNT group. DIA, diacerein; GNT, gentamicin; TLR4, Toll‐like receptor 4.

The GNT group showed a significant increase in parotid TLR4 and C caspase‐3 expressions when compared to the control and DIA groups. Meanwhile, when compared to the GNT group, the GNT+DIA co‐treated rats had a significant decrease in parotid TLR4 and C caspase‐3 expressions (Figure 2A,B). The mean value of the inflammation score was expressed as mean ± SEM (8 rats/group).

### 3.2 Histological examination of the parotid gland

**FIGURE 3:**
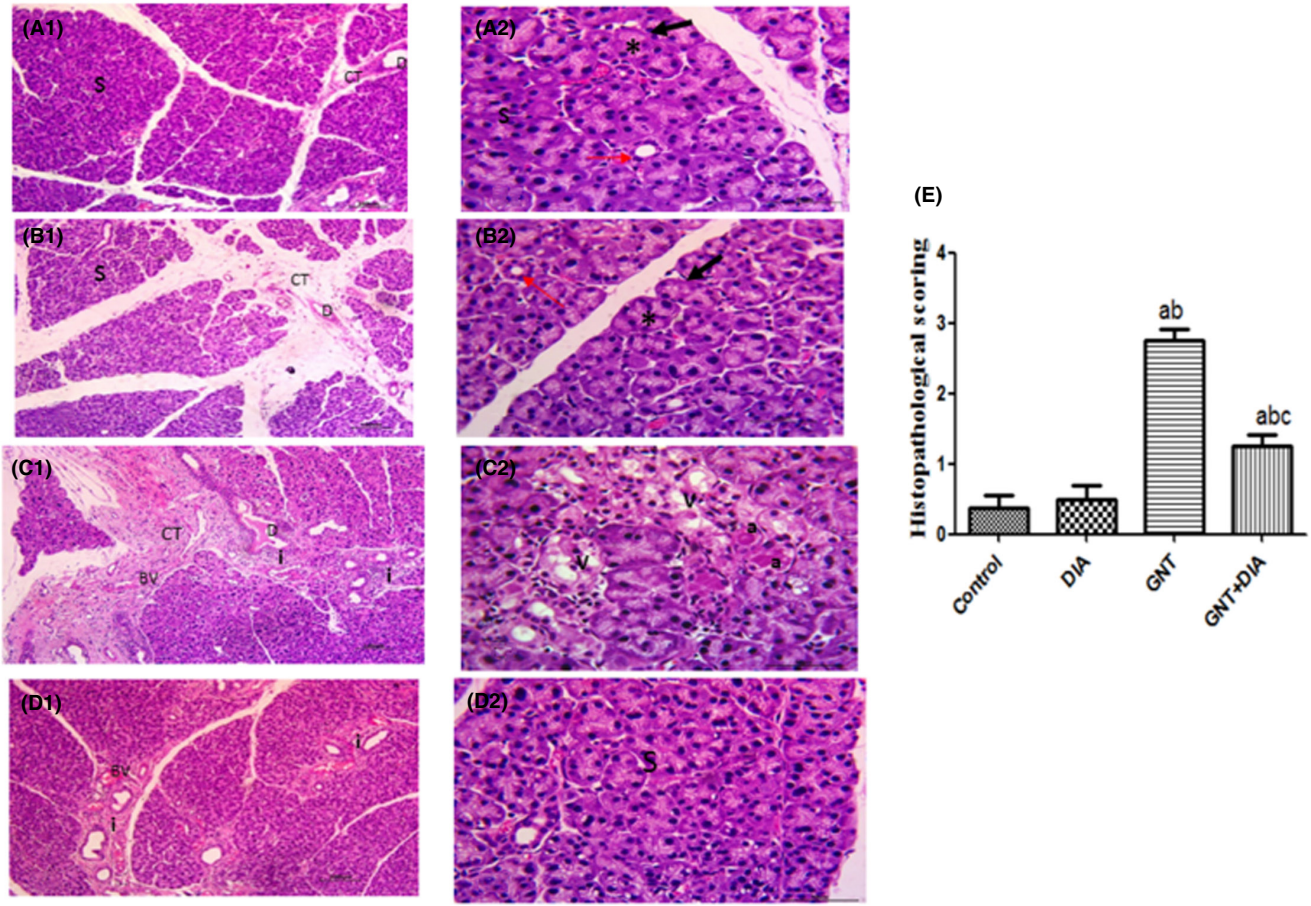
Photomicrographs of haematoxylin‐ and eosin‐stained sections of rat parotid glands of the control (A1 & A2) and DIA (B1 & B2) groups showed parotid gland normal architecture formed of serous acini (S) with basal basophilia (arrow) and apical granular acidophilic cytoplasm (*), intralobular ducts (red arrows) in between and thin connective tissue septa (CT) with interlobular duct (D). The GNT group (C1 & C2) showed thick CT septa (CT) with dilated congested blood vessels (BV), dilated interlobular duct (D) with stagnant secretion and marked inflammatory cell infiltration (i). The acini appear distorted some with vacuolated cytoplasm (V), others showed accumulation of acidophilic homogenous material (A). The GNT+DIA group (D1 & D2) showed some congested blood vessels (BV) and mild inflammatory cells infiltration (i) with normal acini (S) A1, B1, C1, D1 ×100 … A2, B2, C2, D2 ×400. The histopathological inflammatory scoring expressed as mean ± SEM (8 rats/group). ^a^Significant (*p* < 0.05) difference from the control group. ^b^Significant (*p* < 0.05) difference from the DIA group. ^c^Significant (*p* < 0.05) difference from the GNT group done by GraphPad Prism (version 5.0). DIA, diacerein; GNT, gentamicin.

Sections of the parotid in the control and DIA groups had normal stroma and parenchyma of acini and ducts. In contrast, those of the GNT group showed abnormal morphology. The CT septa were thickened with markedly dilated, congested blood vessels and interlobular ducts with stagnant secretion. Marked inflammatory cell infiltration was also noticed. Their acini showed variable morphology. Some appeared distorted with vacuolated cytoplasm, while others demonstrated luminal accumulations of acidophilic homogenous material. Meanwhile, sections of the GNT+DIA group appeared more or less normal despite the presence of mildly congested blood vessels and mild inflammatory cell infiltration. The mean value of the inflammation score and the statistical analysis of it were expressed as mean ± SEM (8 rats/group) (Figure 3).

**FIGURE 4:**
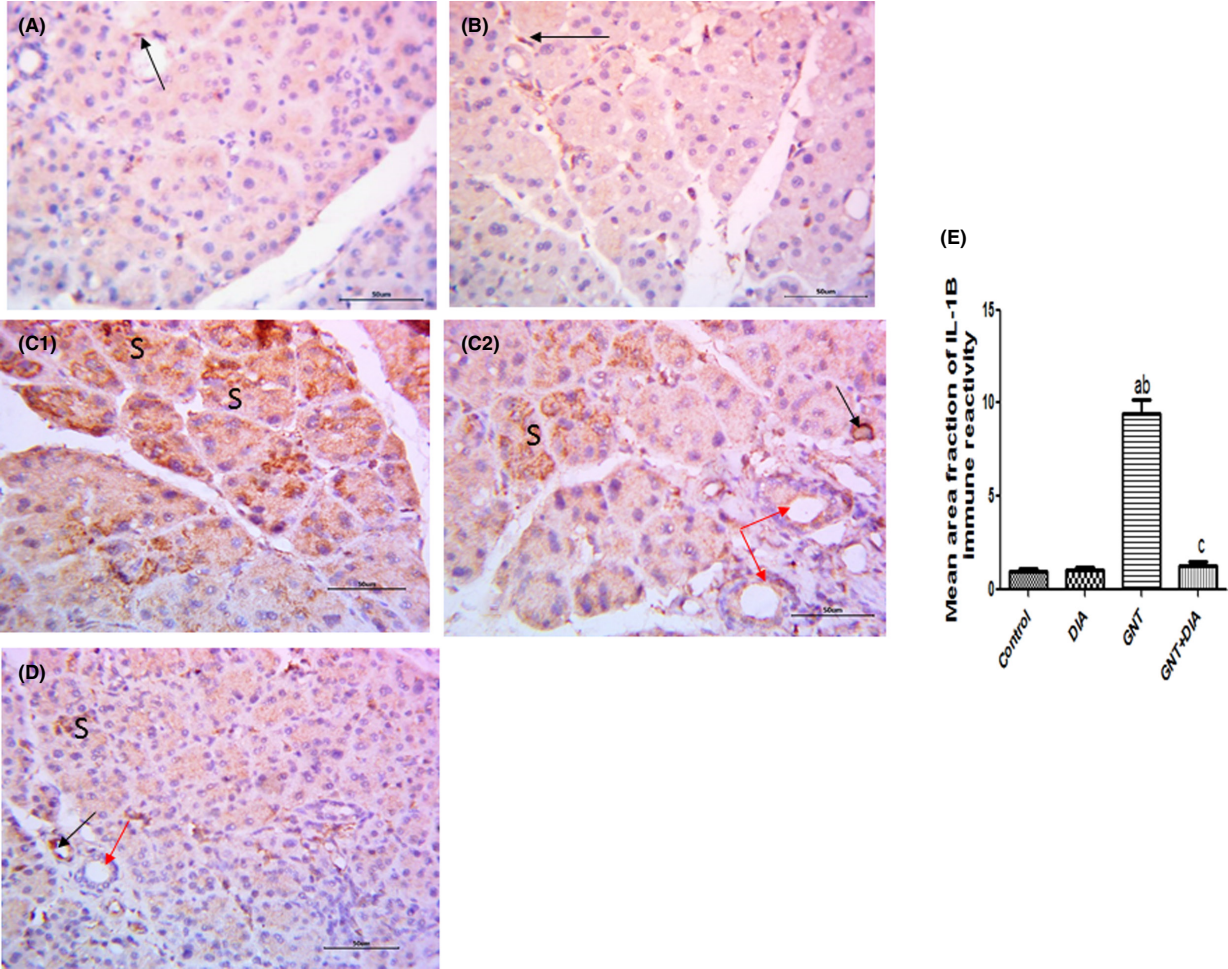
Photomicrographs of sections of rat parotid glands immunostained for IL‐1b of (A & B) (control & DIA groups) showed negative expression except for scattered connective tissue cells in between the acini (arrows). (C1 & C2) The GNT group showed high expression in the acini (S) and in the endothelium lining the blood vessel (black arrow). Notice faint expression in the cells lining the ducts (red arrows). (D) The GNT + DIA group showed faint expression in some acini (S) and in the endothelium lining the blood vessel (black arrow). Notice negative expression in the duct (red arrow). ×400. The mean area fraction of IL‐1β immune‐positive cells in the studied groups expressed as mean ± SEM (8 rats/group). ^a^Significant (*p* < 0.05) difference from the control group. ^b^Significant (*p* < 0.05) difference from the DIA group. ^c^Significant (*p* < 0.05) difference from the GNT group. DIA, diacerein; GNT, gentamicin; IL‐1β, interleukin‐1 beta.

Immunohistochemical staining for Il‐1β in both the control and DIA groups showed negative expression of the acini, ducts and blood vessels. Only scattered connective tissue cells in between the acini showed positive expression. In the GNT group, positive cytoplasmic expression was noticed in the acini, ducts and endothelium of the blood vessels. Meanwhile, in the GNT+DIA group, a faint expression was observed in some acini and in the endothelium lining the blood vessels, while the ducts appeared negative. Morphometric studies and statistical analysis of the mean area fraction of IL‐1β immune‐positive cells in the studied groups were expressed as mean ± SEM (8 rats/group) (Figure 4).

## 4 DISCUSSION

An antibiotic is a type of antimicrobial substance that is active against bacteria. It is the most important type of antibacterial agent for fighting bacterial infections, and antibiotic medications are widely used in the treatment and prevention of such infections. Gentamycin is an aminoglycoside antibiotic derived from *Micromonospora purpurea* and is used in the treatment of many types of bacterial infections, particularly those caused by Gram‐negative organisms.^18^ Despite its wide clinical use, gentamycin has been reported to cause disturbing toxicity. One of the effects of this toxicity is the effect of GNT on salivary glands.^19^

The findings of this study revealed that GNT caused parotid toxicity through a variety of mechanisms. Damage to the salivary gland is caused by an imbalance between oxidative stress, inflammation, and apoptosis,^20^ which are induced by GNT, as revealed in our results. Our study has obviously detected the ability of gentamycin to induce oxidative stress in rat parotid gland and plasma, as shown by a significant decrease in parotid SOD and serum TAC levels and a significant increase in parotid MDA, an indicator of lipid peroxidation that results in oxidative instability. These findings are in agreement with earlier reports.^21^, ^22^

Superoxide anion (O^−2^) and ROS were produced more readily by GNT, which led to injury to the salivary glands. ROS damage any molecules, primarily the polyenoic fatty acids of cell membranes, through a series of events involving membrane lipid peroxidation and necrosis.^23^ The radical scavenging system, which includes SOD and GSH, helps to stop ROS damage in healthy cells.^24^ This supported our findings because of the high ROS emission and the resulting oxidative instability.

Moreover, it has been reported that aminoglycosides change the activities of antioxidant enzymes such as SOD, CAT, GSH peroxidase and GST in different tissues.^25^ Reduced enzyme activity in the gentamycin group is a generic reaction, not a response unique to one enzyme, which suggests numerous steps of the antioxidant system have compromised performance. It was suggested that tissue damage may be caused by aminoglycoside‐induced free radical production and changes in the activity of antioxidant enzymes.^22^

By cross‐linking with MDA, the increased lipid peroxidation causes the enzymes to become inactive. This results in an increased build‐up of superoxide, H_2_O_2_, and hydroxyl radicals, which may further encourage lipid peroxidation. According to the study by Heeba,^26^ this mechanism has evidence. The decrease in antioxidant enzymes may be caused by their quick depletion and weariness from combating free radicals produced during the progression of parotid damage.

According to the current study, pretreatment with DIA reduced the oxidative stress induced by GNT, as evidenced by an increase in parotid SOD and serum TAC levels and a decrease in parotid MDA levels, which is consistent with earlier studies that reported the proper antioxidant capabilities of DIA.^12^, ^27^ It has been reported that in female rats with estradiol benzoate‐induced endometrial hyperplasia and atypia, DIA lowered the uterine MDA level and elevated the SOD level.^28^

GNT induced an increase in inflammatory markers in the current study, as evidenced by a significant increase in parotid TNF‐α, NF‐κB and caspase‐3 levels in the GNT group when compared to the control and DIA groups. Our findings have been confirmed by histological examination, which revealed marked inflammatory cells infiltration in the parotid gland tissue of the GNT group. Sharma et al.,^29^ also demonstrated the ability of GNT to increase inflammatory mediators. Previous studies, in agreement with our data, reported the correlation of the inflammatory response to GNT in clinical and animal studies.^30^, ^31^ Karimi et al.,^32^ reported that GNT treatment induced a significant systemic inflammation, which may be linked to the associated increase of pro‐inflammatory chemokines and cytokines like TNF‐α and IL‐1.

Previous research reported that ROS causes apoptosis and inflammation in a variety of illnesses, in addition, high levels of NF‐ κB controlled the expression of pro‐inflammatory cytokines such IL‐1β, IL‐6 and TNF‐α.^33^ On the contrary, NF‐κB is a key regulator of the production of numerous immunomodulatory mediators connected to oxidative stress.^34^ The oxidative instability that increases the outflow of proinflammatory cytokines by activating the redox‐sensitive transcription factor is related to the inflammatory response of GNT.^35^

Caspases (Caspase‐3 is a main enzyme in apoptosis) are activated and pro‐apoptotic proteins are upregulated during apoptosis, which is also influenced by intracellular Ca^2+^ levels. Severe Ca^2+^ dysregulation can cause ER stress‐mediated apoptosis in response to a variety of clinical situations. Due to the secretory activity of salivary gland cells and the intricacy of their secretory products that are generated, salivary gland cells are vulnerable to ER stress.^36^ Earlier research has demonstrated that ER stress is triggered in Sjögren's syndrome patients' minor salivary gland epithelial cells. When cellular stressors are present, a signalling cascade is set off, leading to the unfolded protein response, which is essential for restoring cellular homeostasis.^37^

In the current study, DIA decreased TNF‐α, NF‐κB and caspase‐3 levels significantly, matching with Refaie et al.^38^ They are thought to play a significant role in parotid gland injury, and diacerein inhibited their upregulation. In line with current research, Pasin et al.^39^ found that diacerein reduced peritoneal fluid TNF‐α and IL‐1β levels and protected young rats from Baker's yeast‐induced fever.

Moreover, the present result showed a significant increase in parotid TLR4 expression in the GNT group. This finding is in accordance with Pakfetrat et al.^34^ They revealed that TLR‐4/ NF‐κB pathway activation plays a significant part in the renal damage caused by GNT. In the triggering of inflammatory reactions, TLR4 is crucial. The nuclear transcription factor NF‐κB, which mediates the production of proinflammatory cytokines including TNF‐α and IL‐1β, can be activated by the TLR4 signalling pathway.^12^

Furthermore, the current study showed that GNT triggers an inflammatory response by upregulating IL‐1β immunoexpression. Our results reported positive cytoplasmic expression of IL‐1β in the acini, ducts and endothelium of the blood vessels of the GNT group. IL‐1β is a key player in the chain of inflammatory events during reperfusion. Leucocytes are drawn to inflammatory regions by chemoattractants like IL‐1β.^40^

Besides, DIA treatment, in this study, suppressed the activation of the TLR4/NF‐κB mediated signalling pathway with consequent reduction of the inflammatory cytokines production of TNF‐α and IL‐1β in parotid tissue and these results are consistent with Oliveira et al.^41^ It has been reported that IL‐1β synthesis is mostly inhibited by DIA. The main inhibitor of the secretion and activity of the IL‐1β cytokine is the IL‐1‐converting enzyme, which is suppressed by DIA. Additionally; it reduces the specific receptor of IL‐1β, and hence prevents IL‐1β attaching to its receptor. Moreover, Rhein, a diacerein active metabolite, reduces IL‐1 production both in vitro and in vivo.^42^

Normal cell processes are critically dependent on the transport of ions across the cell membrane. The Ca^2+^ ion is the common second messenger that controls a wide range of crucial processes in practically all cell types, including gene expression and cellular homeostasis. A channel known as TRPC1 controls the salivary gland's function. It has a role in controlling Ca^2+^ homeostasis. Damage to TRPC1 lowers the ER Ca^2+^ level, which leads to the loss of salivary gland cells and a rise in the expression of CHOP.^43^ The present data showed a significant decrease in parotid TRCP1 level coupled with a significant increase in parotid CHOP level in the GNT group when compared to the control and DIA groups.

It has been reported that TRPC1 controlled the sustained Ca^2+^ entry necessary for TMEM16a (CaCC) activation, which is essential to govern Cl^−^ efflux, to control the Ca^2+^‐activated chloride (Cl^−^) channels (CaCC) in the salivary gland cells. Furthermore, ER and Ca^2+^ homeostasis disorders coexist with illnesses and injuries to the salivary glands. With this damage, CHOP was activated, which decreased TRPC1 expression and decreased autophagy and apoptosis, which led to cell death.^8^

In addition to the antioxidant, anti‐inflammatory and anti‐apoptotic activity of DIA, our results revealed that DIA dramatically elevated parotid TRPC1 level and lowered parotid CHOP level as compared to the GNT group. This was accomplished by reducing the abnormalities in ER and Ca^2+^ homeostasis, which in turn resulted in the deactivation of CHOP and an increase in TRPC1 levels. These results have been confirmed histologically, which revealed a quite normal appearance of parotid gland tissue in the DIA‐treated group.

In conclusion, the results of the present research revealed the efficacy of DIA in reducing oxidative stress and the inflammatory response induced by GNT. DIA downregulated the TLR4/NF‐κB mediated signalling pathway, suppressed IL‐1β expression, and restored parotid gland tissue. Through the antioxidant, anti‐inflammatory and anti‐apoptotic functions of DIA, it ameliorated the signalling mechanisms and saved the parotid salivary gland from GNT‐induced harm.

## AUTHOR CONTRIBUTIONS

**Dalia Mohamed Ali:** Investigation (equal); methodology (equal). **Rehab Ahmed Rifaai:** Methodology (equal); resources (equal). **Michael Atef Fawzy:** Data curation (lead); investigation (equal); methodology (equal). **Medhat Atta:** Investigation (equal); methodology (equal). **Nermeen N. Welson:** Formal analysis (equal); writing – review and editing (lead). **Gaber El‐Saber Bathiha:** Funding acquisition (equal); project administration (lead). **Athanasios Alexiou:** Funding acquisition (equal). **Marios Papadakis:** Funding acquisition (equal). **Walaa Yehia Abdelzaher:** Formal analysis (equal); writing – original draft (lead). **Mohamed Mahmoud:** Funding acquisition (equal).

## FUNDING INFORMATION

This work was supported by the University of Witten‐Herdecke Germany.

## CONFLICT OF INTEREST STATEMENT

The authors have no conflict of interest to declare.

## PATIENT CONSENT STATEMENT

Not applicable.

## PERMISSION TO REPRODUCE MATERIAL FROM OTHER SOURCES

Not applicable.

## CLINICAL TRIAL REGISTRATION

Not applicable.

## Data Availability

The data analysed during this study are available from the corresponding author upon reasonable request.
